# A Novel Immunochromatographic Strip Based on Latex Microspheres for the Rapid Detection of North American-Type Porcine Reproductive and Respiratory Syndrome Virus

**DOI:** 10.3389/fmicb.2022.882112

**Published:** 2022-04-29

**Authors:** Wansheng Li, Minhua Li, Hongliang Zhang, Chao Li, Hu Xu, Bangjun Gong, Jun Fu, Zhenyang Guo, Jinmei Peng, Guohui Zhou, Zhijun Tian, Qian Wang

**Affiliations:** ^1^State Key Laboratory of Veterinary Biotechnology, Harbin Veterinary Research Institute, Chinese Academy of Agricultural Sciences, Harbin, China; ^2^Beijing IDEXX Yuanheng Laboratories, Co., Ltd., Beijing, China

**Keywords:** North American-type PRRSV, latex microsphere, immunochromatographic strip, rapid detection, N protein, monoclonal antibody

## Abstract

A latex microsphere-based immunochromatographic strip (ICS) test was successfully developed for the rapid and sensitive detection of porcine reproductive and respiratory syndrome virus (PRRSV). The PRRSV N protein-specific monoclonal antibody (mAb) 1H4 labeled with latex microspheres was dispensed on a conjugate pad for use as the detector. The same mAb and goat anti-mouse antibody were blotted on a nitrocellulose membrane to generate test and control lines, respectively. The limit of virus detection was approximately 5 × 10^2.0^ median tissue culture infectious dose (TCID_50_)/ml, and the limit of N protein detection was approximately 15 ng/ml. Other common porcine viruses were tested to evaluate the specificity of the ICS, and positive results were observed for only North American-type PRRSV. A comparison of the strip with a standard diagnostic test (reverse transcriptase polymerase chain reaction, RT-PCR) was also performed, and the results showed that the ICS test exhibited relatively high specificity and sensitivity (90.32 and 73.91%, respectively) and relatively high positive predictive value (PPV) and negative predictive value (NPV; 85 and 82.35%, respectively). These results suggest that the ICS test can be used to rapidly and accurately detect PRRSV and can be suitable for diagnostic applications in the field.

## Introduction

Porcine reproductive and respiratory syndrome (PRRS) is one of the most economically important infectious diseases of swine throughout the world. This virus was first reported in 1987 in the United States and was subsequently reported in Europe in 1991 (Wensvoort et al., 1991; Collins et al., 1992). The disease is characterized by reproductive failure in sows and respiratory complications in piglets and growing pigs (Albina, 1997; Rossow, 1998). The etiologic agent of PRRS has been identified as porcine reproductive and respiratory syndrome virus (PRRSV), which has been disturbing the worldwide swine industry for the past 30 years and has led to staggering economic losses (Pejsak et al., 1997; Neumann et al., 2005; Zhou and Yang, 2010).

PRRSV is an enveloped, positive, single-stranded RNA virus of the genus *Porartevirus* that belongs to the family *Arteriviridae* within the order *Nidovirales* (Adams et al., 2017). PRRSV can be divided into two species: genotype I, represented by the prototypical European strain Lelystad virus (LV; Wensvoort et al., 1991), and genotype II, represented by the North American strain ATCC VR-2332 (Benfield et al., 1992; Adams et al., 2017). In China, most isolated strains are of genotype II (Chen et al., 2006; Xu et al., 2020). The PRRSV genome has a length of 15 kb and contains at least 10 open reading frames (ORFs). ORF1a and ORF1b encode viral replicase polyproteins, which are translated immediately upon viral entry and are then proteolytically processed by virally encoded proteinases into 16 mature non-structural proteins (NSP1a, NSP1b, NSP2-NSP6, NSP2TF, NSP2N, NSP7a, NSP7b, and NSP8-NSP12; Fang and Snijder, 2010; Fang et al., 2012; Li et al., 2014). The structural protein-coding region of PRRSV encodes eight structural proteins: GP2, E, GP3, GP4, GP5, M, N, and GP5a (Meulenberg et al., 1995b; Bautista et al., 1996; Johnson et al., 2011). The N protein is a highly immunogenic protein and the most abundant viral protein expressed in infected cells (Meulenberg et al., 1995a,b; Loemba et al., 1996; Dea et al., 2000), which makes it a suitable candidate for the detection of virus-specific antibodies and disease diagnosis (Bogdanova et al., 2007; Wei et al., 2020).

Several diagnostic methods, including virus isolation assays (Wensvoort et al., 1991), reverse transcriptase polymerase chain reaction (RT-PCR; Spagnuolo-Weaver et al., 1998), and quantitative real-time PCR (Park et al., 2019), have been used for the detection of PRRSV antigens. Although these laboratory tests offer good sensitivity and specificity in virus detection, some shortcomings limit their application; for example, the operation process is time-consuming, and special or expensive equipment and professionally trained technicians are needed. The immunochromatographic strip (ICS) method has the advantages of relatively high sensitivity, simple operation, short time requirement, minimal labor requirement, and low cost and is suitable for the rapid on-site screening of a large number of samples. In recent years, the ICS method has been rapidly developed in the field of veterinary drug residue detection and disease diagnosis (Li et al., 2019b; Liu et al., 2019; Wang et al., 2019; Wei et al., 2020; Di Nardo et al., 2021).

The ICS test for detecting PRRSV antibodies or antigens using colloidal gold as the tracer has been reported (Zhou et al., 2009; Li et al., 2017; Yu et al., 2018). However, an immunochromatographic assay based on latex microspheres as the tracer for the detection of PRRSV has not been described. The aim of this study was to establish a rapid, sensitive, and specific technology for the detection of North American-type PRRSV (NA-PRRSV) antigens during the early period of PRRSV infection, and an innovative ICS was developed using latex microspheres as the labeling probe instead of gold nanoparticles.

## Materials and Methods

### Animal Study Ethics Statements

This study was conducted in accordance with the recommendations provided in the Guide for the Care and Use of Laboratory Animals of the Ministry of Science and Technology of China. The Committee on the Ethics of Animal Experiments of the Harbin Veterinary Research Institute (HVRI) of the Chinese Academy of Agricultural Sciences (CAAS) reviewed and approved the protocols. Six-week-old BALB/c mice were provided by Beijing Weitong Lihua Laboratory Animal Technology Co., Ltd. (experimental animal license number: SYXK (Jing) 2012–0024). PRRSV immunization experiments using these 6-week-old BALB/c mice were conducted within the animal biosafety level 2 facilities of the HVRI of the CAAS (approval number SY-2020-MI-123).

### Viruses and Reagents

The HP-PRRSV strain HuN4, HP-PRRSV vaccine (HuN4-F112, TJM-F92, JXA1-R), classical PRRSV vaccine CH-1R and MLV, NADC30-like PRRSV SDHS53, NADC34-like PRRSV TZJ1341, European PRRSV (VP046 BIS and DV) and recombinant SDHS53 N protein (240 μg/ml) used in this study were retained at the HVRI of the CAAS. Porcine parvovirus (PPV), porcine circovirus type 2 (PCV-2), classical swine fever virus (CSFV), transmissible gastroenteritis virus (TGEV), porcine pseudorabies virus (PRV), and porcine epidemic diarrhea virus (PEDV) were collected at the HVRI. Latex microspheres (diameter = 300 nm) were purchased from Hangzhou Xiaoque Technology Co., Ltd. (Hangzhou, China). MES, EDC·HCl, and NHS were procured from Sigma (Sigma, St. Louis, MO, United States). Nitrocellulose (NC) membranes (Millipore 180) were obtained from Millipore (Billerica, United States). Polyester membranes were purchased from Pall Corporation (NY, United States), and absorbent paper and PVC sheets were procured from Shanghai Jinbiao Biotechnology Co., Ltd. (Shanghai, China).

### Production and Characterization of mAbs

Female BALB/c mice aged 6 weeks were immunized intraperitoneally three times at 2 week intervals with SDHS53 (10^7.0^ median tissue culture infectious dose (TCID_50_)/ml) in Freund’s complete adjuvant (Sigma, St. Louis, MO, United States) for the first immunization and in Freund’s incomplete adjuvant for the other two immunizations and then boosted intraperitoneally with virus only. Three days after the final booster injection, the mice were euthanized by carbon dioxide asphyxiation, and spleen cells were then fused with SP2/0 cells using 50% (v/v) polyethylene glycol (Sigma, St. Louis, MO, United States). The fused cells were successively cultured in Dulbecco’s modified Eagle’s medium (DMEM; Gibco BRL Co., Ltd., United States) containing HAT (Sigma, St. Louis, MO, United States), in DMEM containing HT (Sigma, St. Louis, MO, United States) and then in DMEM supplemented only with 20% fetal bovine serum (HyClone Laboratories Inc., South Logan, UT, United States). Hybridoma cell lines secreting antibodies against PRRSV were screened and subcloned at least three times by limiting dilution. The hybridoma culture supernatants were screened for antibodies using an indirect immunofluorescence assay (IFA). Antibodies that bound to PRRSV but failed to bind MARC-145 cells were considered positive for PRRSV. Stable cells were injected into the abdominal cavities of BALB/c mice that had been pretreated with Freund’s incomplete adjuvant. Approximately 1 week later, the ascites were harvested, and the mAbs were purified from the ascites using an antibody purification kit (Protein G Resin, GenScript, Nanjing, China) per the manufacturer’s instructions. The isotype of the produced mAb was determined with a Pierce Rapid ELISA Mouse mAb Isotyping Kit (Thermo Scientific, MA, United States) according to the manufacturer’s instructions.

Transient transfection was performed to identify the structural proteins that bound to the mAbs. 293T cells were transiently transfected with the eukaryotic expression vector p3 × FLAG-CMV-SDHS53-GP2, GP3, GP4, GP5, M, or N using X-tremeGENE HP DNA Transfection Reagent (Roche, Basel, Switzerland) according to the manufacturer’s protocol. p3 × FLAG-CMV-transfected cells were used as a negative control. The cells were harvested, and IFA was performed as described above.

### Localization of the B-Cell Linear Epitope Using Overlapping SDHS53-N Protein Fragments

The ORF7 gene of SDHS53 was divided into two overlapping fragments: N1 and N2. Specific primers (Table 1) were used to amplify these fragments. The PCR products amplified from these two fragments were cloned separately into the pGEX-6p-1 expression vector and used to transform E. coli BL21 (DE3) cells (Tiangen, Beijing, China) in which the corresponding proteins were expressed. The recombinant proteins were identified by SDS-PAGE and Western blotting as described above. Upon the identification of bound polypeptides, N2 of SDHS53 was divided into several segments with N-terminal truncations, and these fragments were identified by SDS-PAGE and Western blotting as described above.

**Table 1:** Primer sets for amplification of the SDHS53 ORF7 gene and its truncated fragments.

Name of fragment	Sequences of PCR primers (5′-3′)	Positions of nucleotide acids in ORF7	Positions of amino acids in N protein
N1	F: CGCGGATCCATGCCAAATAACAACGGC	1–222	1–74
	R: CCGCTCGAG**TCA**CAATTGTCGCTCACTAGG		
N2	F: CGCGGATCCCCTCTAGCGACTGAAGAT	166–372	56–123
	R: CCGCTCGAG**TCA**TGCTGAGGGTGACG		
N21	F: CGCGGATCCGACGTCAGACATCACTTC	184–372	62–123
N22	F: CGCGGATCCACCCCTAGTGAGCGACAA	202–372	68–123
N23	F: CGCGGATCCTTGTGTCTGTCGTCAATCC	220–372	74–123
N24	F: CGCGGATCCCGGACTGCCTTTAACCAA	238–372	80–123
N25	F: CGCGGATCCGGCGCTGGAACTTGCAC	256–372	86–123
N26	F: CGCGGATCCCTGTCAGACTCAGGGAGA	274–372	92–123
N27	F: CGCGGATCCCCTACTCATCATACCGT	319–372	107–123
N28	F: CGCGGATCCCGCCTGATTCGCGCCACA	337–372	113–123
N29	F: GATCCCGCGCCACAGCGTCACCCTCAGCA**TGA**C	346–372	116–123
	R: TCGAG**TCA**TGCTGAGGGTGACGCTGTGGCGCGG		
N30	F: GATCCGCGTCACCCTCAGCA**TGA**C	355–372	119–123
	R: TCGAG**TCA**TGCTGAGGGTGACGCG		

In these primer sets, the forward and reverse primers contain BamH I and Xho I recognition sites, respectively (underlined). The bold font indicates termination codons. The N21 ~ N28 fragments used the same reverse primers as the N2 fragments. The last two fragments (N29 and N30) were obtained by directly annealing oligomeric nucleic acid fragments, which also contain BamH I and Xho I recognition sites (underlined).

### Multiple Sequence Alignment and Spatial Structure Localization of the Epitope on the SDHS53-N Protein Recognized by the mAb

The amino acid sequences of representative PRRSV N proteins (classical PRRSV, HP-PRRSV, lineage 3 PRRSV, NADC30-like strains, NADC34-like strains, and EU-PRRSV) were aligned using DNASTAR MegAlign software. Protein structure models of the SDHS53-N protein were predicted using the SWISS-MODEL online website.^1^ The three-dimensional (3D) structure data were analyzed using PyMOL software (Hu et al., 2016).

### Preparation of a Latex Microsphere-mAb Conjugate and Construction of an ICS

The optimal antibody concentrations for the test lines, control lines, and conjugation with the latex microsphere solution were determined as previously described (Shi et al., 2021), with some modifications. First, the latex microspheres were activated. Briefly, the latex microspheres were washed twice in double-distilled water, and 0.9 ml of 0.1 mol/l MES (pH 6.5), 0.1 ml of 20 mg/ml EDC·HCl, and 0.075 ml of 20 mg/ml NHS were then added successively to each 0.2 ml of latex microspheres. The mixture was shaken for 2 h and then centrifuged at 8000 × *g* for 10 min. The precipitate was resuspended in 0.1 mol/l boric acid buffer (pH 7.2), and the mixture was shaken for 15 min after ultrasonic homogenization. This washing procedure was repeated twice.

Subsequently, 0.5 ml of activated latex microspheres was mixed with 0.5 ml of N protein-specific mAb 1H4, shaken for 2 h, and then centrifuged at 10000 × *g* for 25 min. The precipitate was resuspended using 1 ml of 0.5% (w/v) casein to remove potential excess reactivity. The mixture was shaken for 15 min after ultrasonic homogenization. This blocking procedure was repeated twice.

**Figure 1:**
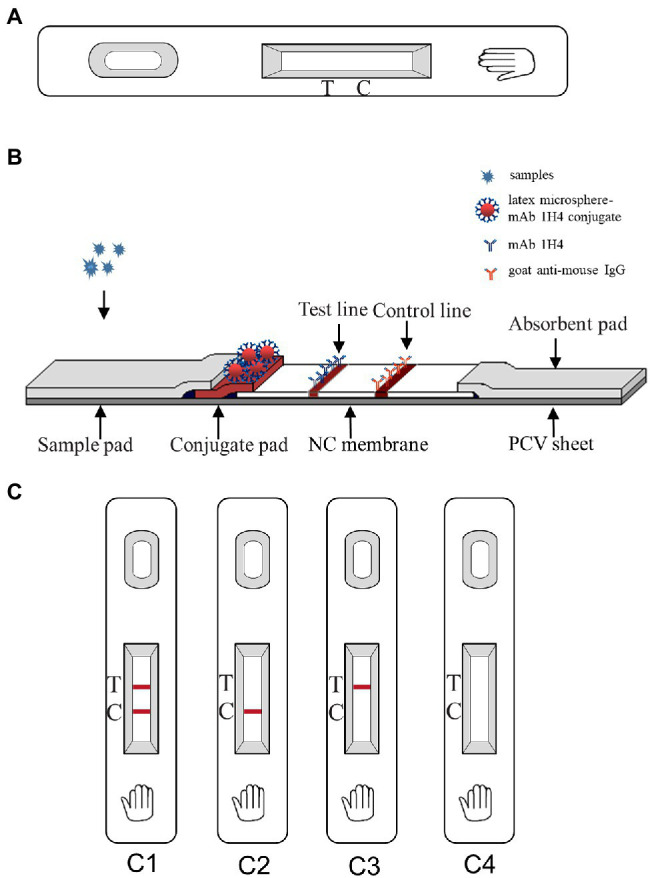
Schematic diagram of the immunochromatographic strip (ICS) and interpretation of the results. **(A)** Outside view of the ICS; **(B)** Inside view of the ICS; **(C)** Interpretation of the ICS result. C1 indicates a positive result; C2 indicates a negative result; and C3 and C4 indicate an invalid test.

The ICS (Figure 1A) includes four components: a sample pad, a conjugate pad, an NC membrane, and an absorbent pad (Figure 1B). The NC membrane was incubated with two antibodies, mAb 1H4 and goat anti-mouse IgG, dissolved in PBS to generate the test and control lines, respectively. The conjugate pad, which is composed of a polyester membrane, was treated with the latex microsphere-mAb 1H4 conjugate by spraying using an XYZ3050 Dispense Workstation at 3 μl/cm and then dried for 1 h at 37°C. mAb 1H4 and goat anti-mouse IgG were diluted with PBS to final concentrations of 2.0 and 1.0 mg/ml, respectively. The XYZ3050 Dispense Workstation (BioDot, Inc., Sky Park, Irvine, CA, United States) was used to spray both antibodies, and the NC membrane was then dried for 1 h at 37°C.

### Assembly Principle and Use of the Test Strips

The test strips were assembled and used as described by Wang et al. (2019). The sample pad, conjugate pad, NC membrane, absorbent pad, and PVC sheet were assembled in sequence. The sample pad and the conjugate pad overlapped by 2 mm with one end of the conjugate pad; the other end of the conjugate pad overlapped (2 mm) with one end of the NC membrane underneath the conjugate pad; and the absorbent pad overlapped by 2 mm with the other side of the NC membrane. Subsequently, the whole plate was cut into 3-mm-wide strips, and these strips were assembled in strip cassettes with desiccant.

During the testing process, 100 μl of liquid sample was dropped onto the sample pad. When the sample liquid reached the conjugate pad, PRRSV reacted with the latex microsphere-1H4 conjugate to form an antigen-latex microsphere-1H4 complex. The complex then traveled through the NC membrane *via* capillary action and then reacted with mAb 1H4 on the test line, resulting in a dark red band. Conversely, in samples lacking PRRSV, the free conjugate that could not bind to the sample continued to travel to the control line. At the control line, the goat anti-mouse IgG antibody reacted with mAb 1H4 on the complex, and a dark red band appeared. The results could be observed within 8 min at room temperature. Two bands appeared with the positive samples (one on the test line and one on the control line), whereas only one band appeared (on the control line) with the negative samples, and no appearance of a band indicated that the test was invalid (Figure 1C).

### Specificity, Sensitivity, and Applicability of the ICS

Common porcine viruses, including EU-PRRSV, PPV, PCV-2, CSFV, TGEV, PRV, and PEDV, were tested to evaluate the specificity of the ICS. PRRSV HuN4 and SDHS53 were used as positive controls. All virus types were used at the same dose (2 × 10^4.0^ TCID_50_/ml).

To evaluate the sensitivity of the ICS, 10^6.0^ TCID_50_/ml PRRSV HuN4 and recombinant SDHS53 N protein (240 μg/ml) were serially diluted in PBST and PBS, respectively, and 100 μl of each dilution was used for the ICS test. The sensitivity was determined by finding the minimum dilution concentration that yielded a positive result. In contrast, the sensitivity of the strip was evaluated by comparing the detection of the same sample with that of PCR using the following primer sequences targeting ORF5: forward 5′-AATGTGTCAGGCGTCGTGGCT-3′ and reverse 5′-CAGAATGTACTTGCGGCCTAGC-3′. In brief, DMEM was used to dilute HuN4 (10^6.0^ TCID_50_/ml) from 10^−1^ to 10^−6^, and viral RNA was extracted from 140 μl of the solution using a TIANamp Virus RNA Kit (Tiangen, Beijing, China). The ORF5 gene containing its flanking regions was amplified using the abovementioned primers. Thermal cycling was performed as follows: one cycle of 95°C for 5 min; 30 cycles of 94°C for 30 s, 55°C for 1 min, and 72°C for 1 min 30 s; and a final cycle of 72°C for 10 min. The PCR products were electrophoresed on a 1% agarose gel. The PCR product (1,107 bp) was sequenced by Comate Bioscience Co., Ltd. (Changchun, China).

To evaluate the applicability of the ICS, HuN4, HuN4-F112, TJM-F92, JXA1-R, CH-1R, MLV, SDHS53, TZJ1341, VP046 BIS, and DV were used for the ICS test.

### Reproducibility and Stability of the ICS

To ascertain the reproducibility and stability of the ICS during storage at room temperature (RT), 3 batches of strips (batch numbers: 202101, 202102, and 202103) were used for the detection of PRRSV (10 virus samples were used to evaluate the applicability of the ICS were selected). All strips from each batch were stored at RT and used for two tests of the samples at a 6-month interval.

### Clinical Application of the ICS

One hundred eight clinical serum and lung tissue samples from pigs with suspected PRRSV infection in Northeast China were collected and tested for PRRSV using the ICS test and PCR. The coincidence rate of the two methods was then calculated.

## Results

### Development and Identification of the mAb

**Figure 2:**
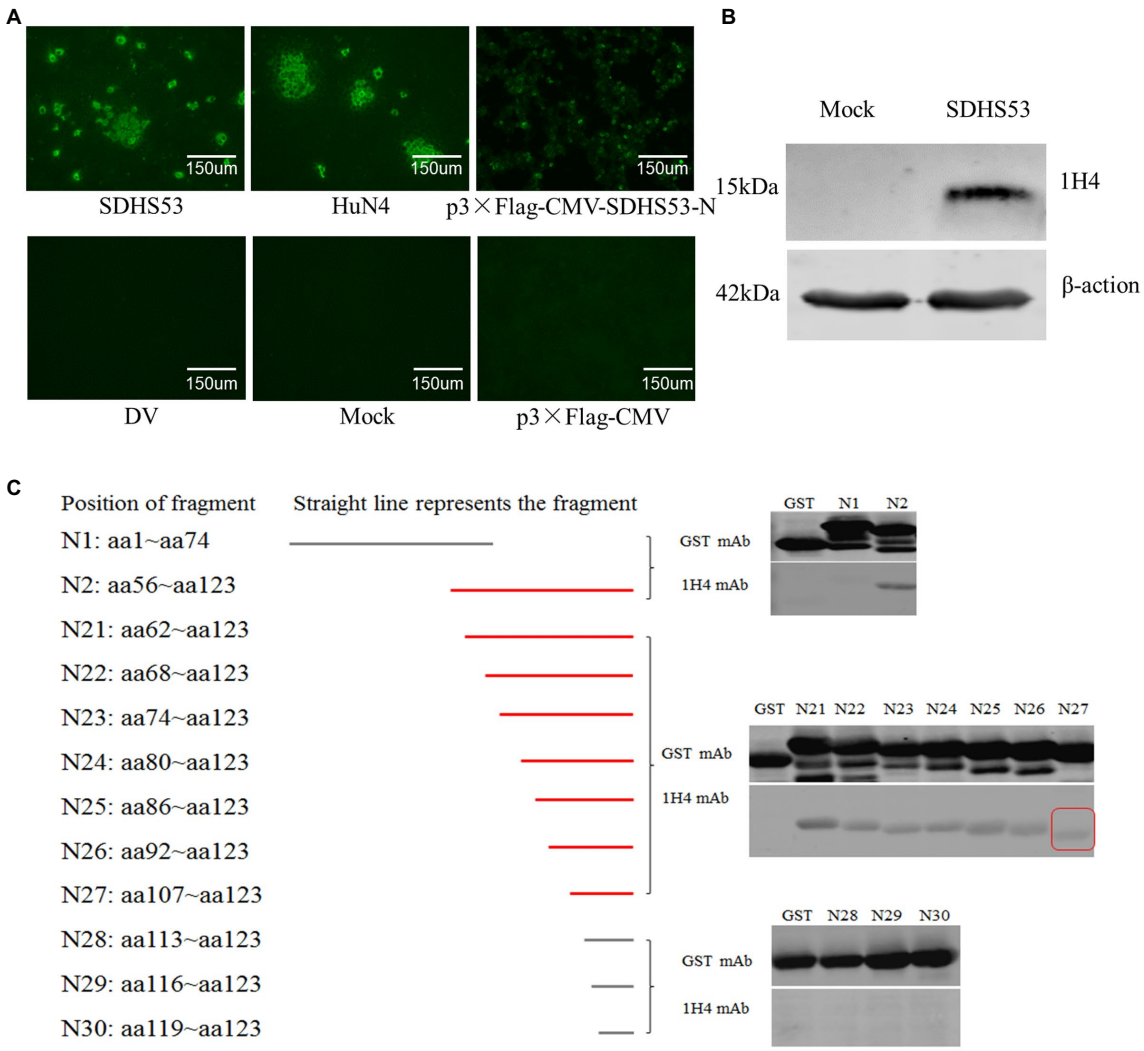
Identification of mAb 1H4 that reacted with SDHS53 N protein. **(A)** Reactivity of mAb 1H4 with PRRSV and its N protein determined by IFA. mAb 1H4 reacted with NADC30-like PRRSV SDHS53 in Marc-145 cells and transiently transfected 293 T cells expressing the N protein. **(B)** Reactivity of mAb 1H4 with SDHS53 determined by Western blotting. **(C)** Identification of the epitope recognized by mAb 1H4 by Western blotting. All segments except N29 and N30 were amplified by PCR, whereas segments N29 and N30 were obtained by primer annealing. Peptide binding with mAb 1H4 is indicated by red lines. The smallest peptide binding with mAb 1H4 is indicated by a red box.

One mAb, 1H4, was generated by IFA detection using SDHS53 as the antigen source. The isotype of mAb 1H4 was identified with a Pierce Rapid ELISA Mouse mAb Isotyping Kit as IgG1. mAb 1H4 specifically recognized the N protein expressed by transiently transfected cells (Figure 2A) and not other structural proteins (data not shown). Western blotting showed that the mAb specifically recognized the 15-kDa protein bands of PRRSV (Figure 2B).

The Western blot and IFA results described above confirmed that mAb 1H4 recognized the N protein. Two overlapping fragments (N1 and N2) from the ORF7 gene of SDHS53 were prepared by PCR and cloned into an expression vector, pGEX-6p-1, for expression as GST fusion proteins. After nucleotide sequencing, the recombinant protein fragments encoded by these constructs were expressed in *E. coli* BL21 (DE3) cells and identified by SDS-PAGE and Western blotting using mAb 1H4. The results showed that mAb 1H4 recognized SDHS53-N2 (aa 56 ~ 123). The N2 fragment was then serially truncated from the N-terminus, and SDHS53-N27 (aa 107 ~ 123) was recognized by mAb 1H4 (Figure 2C).

### Multiple Sequence Alignment and Spatial Location of the Epitope Recognized by mAb 1H4

**Figure 3:**
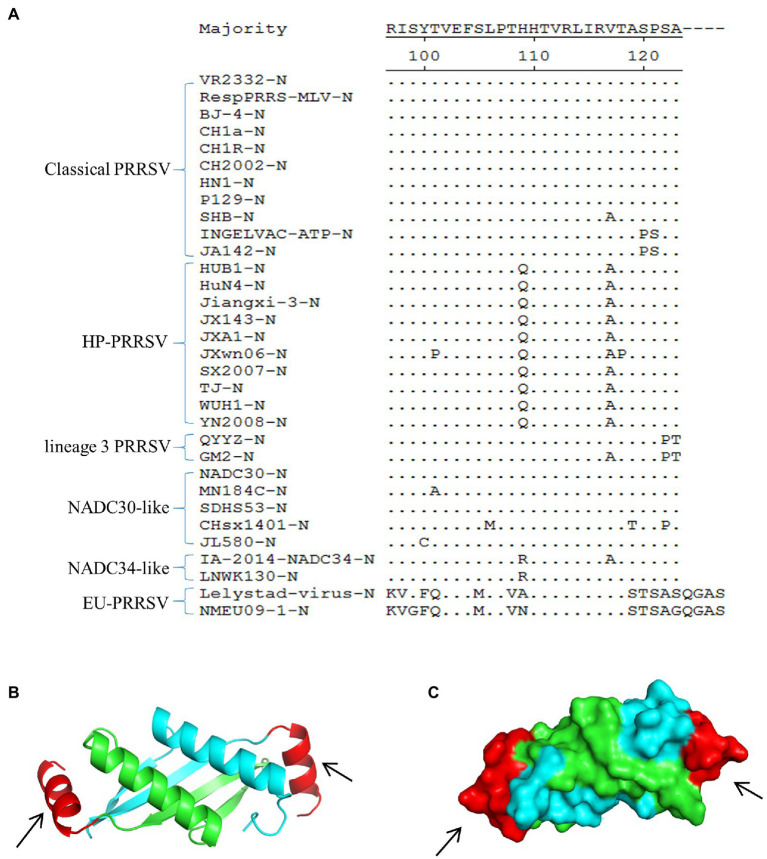
Multiple sequence alignment and spatial localization of the N protein epitope recognized by mAb 1H4. **(A)** Multiple sequence alignment result. The evaluated strains include classical PRRSV, HP-PRRSV, lineage 3 PRRSV, NADC30-like strains, NADC34-like strains, and EU-PRRSV. The epitope on the N protein is highly conserved among the HP-PRRSV strains and classical PRRSV, whereas several substitutions are present in the other PRRSVs. The amino acid sequences were aligned using DNASTAR MegAlign software. **(B)** 3D structure of the SDHS53-N protein (aa 68 ~ 118) predicted using the SWISS-MODEL online server. Different colors represent each monomer of the homodimer N protein, and the mAb 1H4 epitope (aa 107 ~ 123) is highlighted in red (aa 107 ~ 118). **(C)** The relative localizations of the identified mAb 1H4 epitope on the predicted 3D structure of SDHS53-N protein are highlighted in red by PyMOL software (the location marked by the black arrow).

Multiple sequence alignment of representative PRRSV N proteins showed that the epitope (^107^PTHHTVRLIRVTASPSA^123^) recognized by mAb 1H4 was highly conserved among the HP-PRRSV strains and classical PRRSV strains, whereas several substitutions were present in the other PRRSVs (Figure 3A). Visualization of the predicted 3D structure, which was based on simulated data from the SWISS-MODEL online server, showed that the C-terminus of the N protein (aa 68 ~ 118) existed in the form of a homodimer (Figure 3B). Subsequently, the 3D structure data were analyzed using PyMOL software, and the results revealed that the epitope (aa 107 ~ 123) recognized by mAb 1H4 was fully exposed on the surface (aa 107 ~ 118) of each N protein monomer (Figure 3C).

### Specificity of the ICS

**Figure 4:**
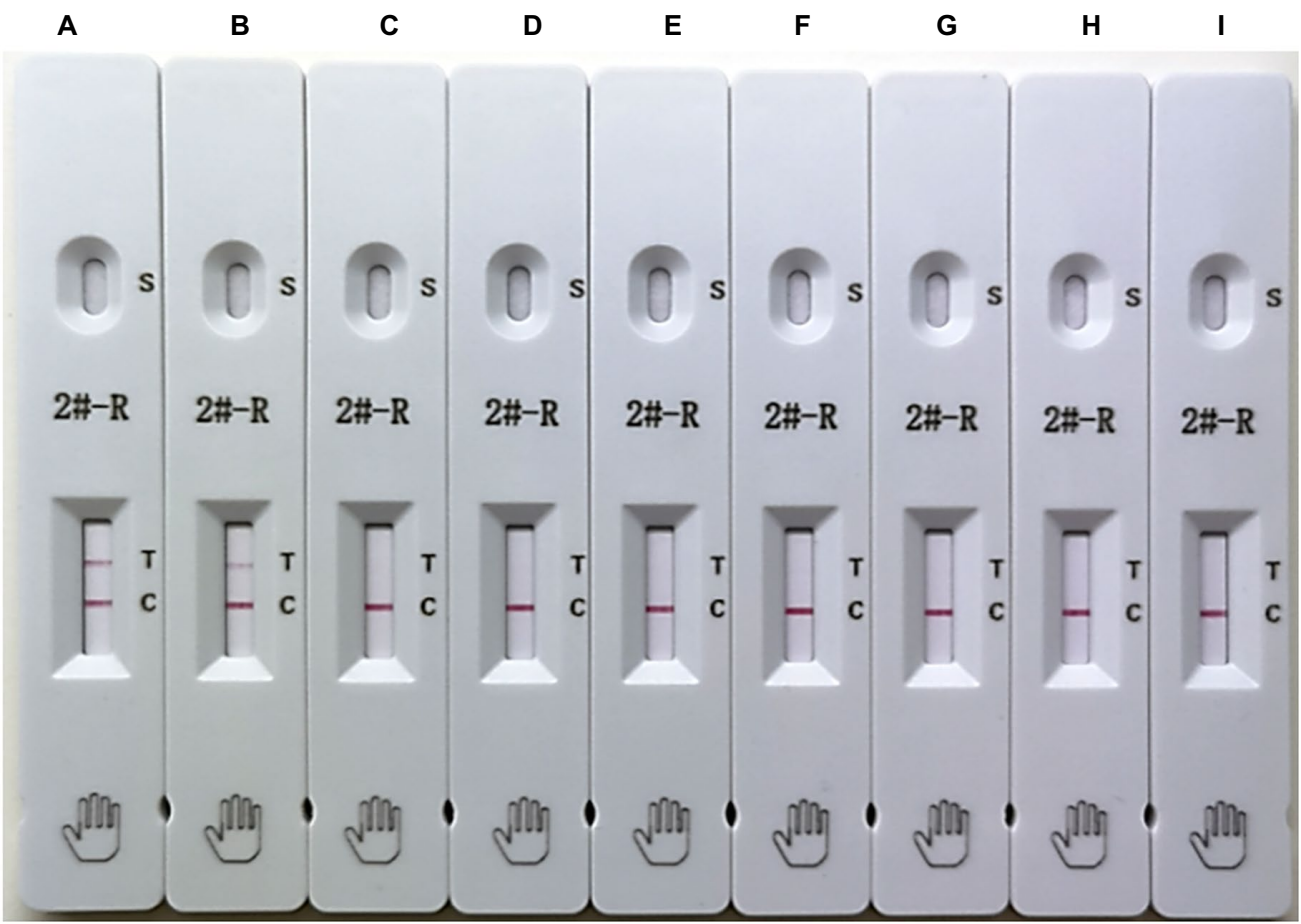
Testing of the specificity of the ICS. The following viruses were tested using the ICS assay developed in this study: HuN4 (HP-PRRSV; strip **A**), SDHS53 (NADC30-like PRRSV; strip **B**), DV (EU-PRRSV; strip **C**), porcine parvovirus (PPV; strip **D**), porcine circovirus type 2 (PCV-2; strip **E**), classical swine fever virus (CSFV; strip **F**), transmissible gastroenteritis virus (TGEV; strip **G**), porcine pseudorabies virus (PRV; strip **H**), and porcine epidemic diarrhea virus (PEDV; strip **I**).

The assay specificity was determined using EU-PRRSV, PPV, PCV-2, CSFV, TGEV, PRV, and PEDV. As demonstrated by the strips shown in Figure 4, PRRSV was positive at the test line, and the other viruses were negative, which suggested that the ICS exhibited good specificity.

### Sensitivity of the ICS

**Figure 5:**
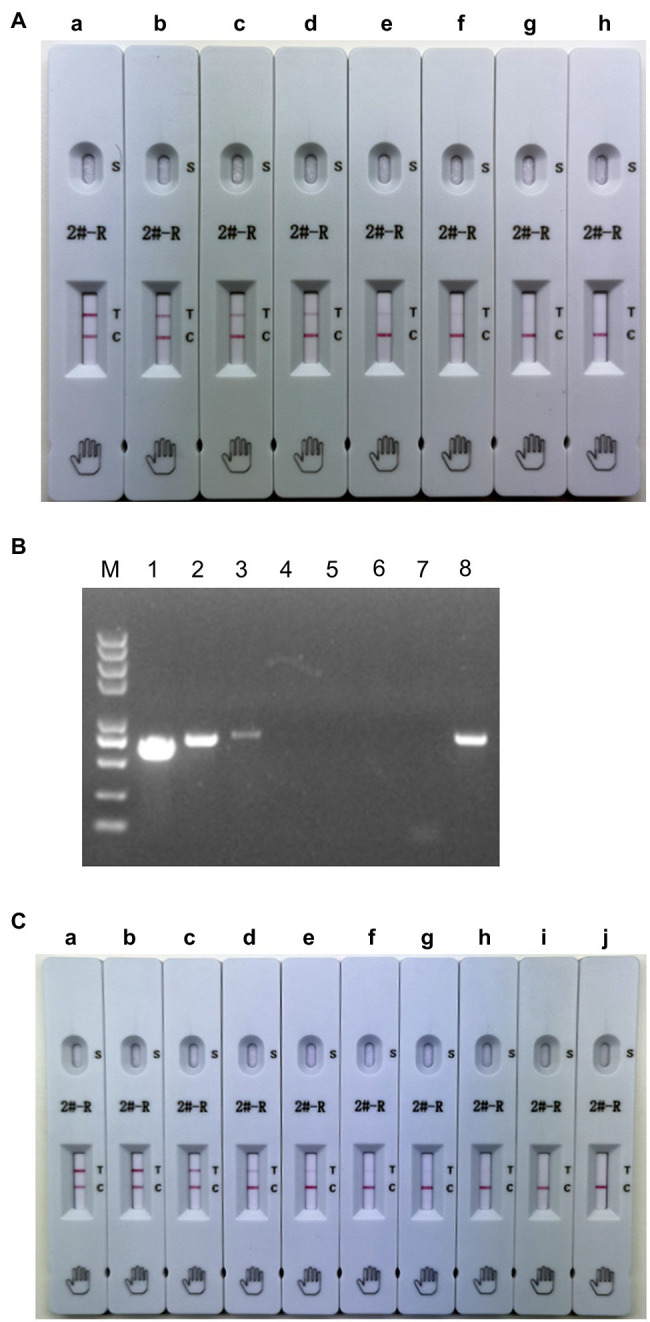
Testing of the sensitivity of the ICS. Serial dilutions of PRRSV HuN4 (10^6.0^ TCID_50_/ml; **A,B**) and recombinant SDHS53-N protein (240 μg/ml; **C**) were prepared in sample dilution buffer and tested using the ICS assay developed in this study and PCR. In **(A)**, the tested virus dilutions were (A) 10^5^ TCID_50_/ml, (B) 10^4.0^ TCID_50_/ml, (C) 5 × 10^3.0^ TCID_50_/ml, (D) 2.5 × 10^3.0^ TCID_50_/ml, (E) 10^3.0^ TCID_50_/ml, (F) 5 × 10^2.0^ TCID_50_/ml, (G) 2.5 × 10^2.0^ TCID_50_/ml, and (H) PBST. In **(B)**, the tested virus dilutions were (1) 10^5.0^ TCID_50_/ml, (2) 10^4.0^ TCID_50_/ml, (3) 10^3.0^ TCID_50_/ml, (4) 10^2.0^ TCID_50_/ml, (5) 10 TCID_50_/ml, (6) 1 TCID_50_/ml, (7) DMEM, and (8) positive control. In **(C)**, the tested dilutions of recombinant N protein were (A) 24,000 ng/ml, (B) 2,400 ng/ml, (C) 240 ng/ml, (D) 120 ng/ml, (E) 60 ng/ml, (F) 30 ng/ml, (G) 15 ng/ml, (H) 7.5 ng/ml, (I) 3.75 ng/ml, and (J) PBS.

To determine the sensitivity of the ICS, serial dilutions of PRRSV HuN4 (10^6.0^ TCID_50_/ml) were prepared in PBST and added to the ICS sample pad. The results showed that the limit of detection of the ICS was 5 × 10^2.0^ TCID_50_/ml (Figure 5A). To evaluate the PCR sensitivity, DMEM was used to dilute HuN4 (10^6.0^ TCID_50_/ml) from 10^−1^ to 10^−6^, and RT-PCR was performed. The minimum virus amount was 10^3.0^ TCID_50_/ml (Figure 5B). The recombinant SDHS53 N protein (240 μg/ml) was serially diluted in PBST to evaluate the sensitivity of the ICS, and the results showed that the minimum detection limit was 15 ng/ml (Figure 5C). The above results showed that the sensitivity of the ICS was very high at both the TCID_50_ level and the protein level.

### Applicability of the ICS

**Figure 6:**
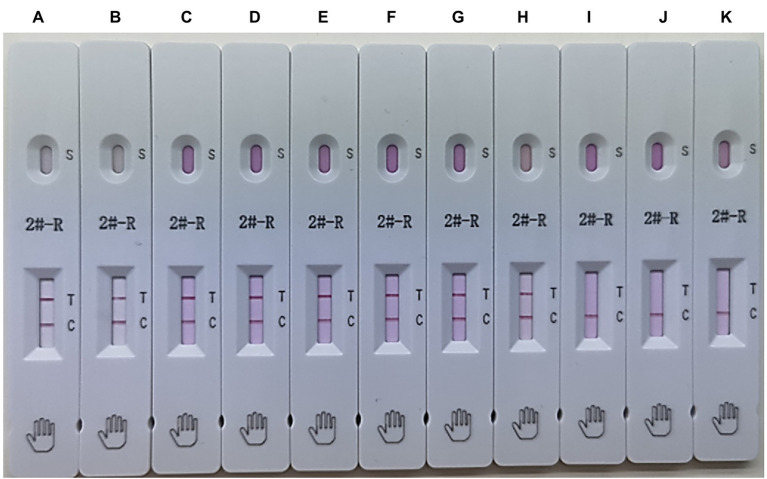
Applicability of the ICS. Several types of PRRSVs were tested using the ICS assay developed in this study: HuN4 (HP-PRRSV; strip **A**), HuN4-F112 (HuN4 vaccine; strip **B**), TJM-F92 (HP-PRRSV vaccine; strip **C**), JXA1-R (HP-PRRSV vaccine; strip **D**), CH-1R (classical PRRSV vaccine; strip **E**), MLV (classical PRRSV vaccine; strip **F**), SDHS53 (NADC30-like PRRSV; strip **G**), TZJ1341 (NADC34-like PRRSV; strip **H**), VP046 BIS (EU-PRRSV vaccine; strip **I**), DV (EU-PRRSV; strip **J**), and control (DMEM; strip **K**).

To evaluate the applicability of the ICS, the ICS test was performed using HuN4, HuN4-F112, TJM-F92, JXA1-R, CH-1R, MLV, SDHS53, TZJ1341, VP046 BIS, and DV. The results showed that the ICS could detect all types of NA-PRRSVs (Figure 6), whereas EU-PRRSVs were not detected, which greatly broadens the applicability of the ICS.

### Reproducibility and Stability of the ICS

To determine its reproducibility and stability, the ICS assay was performed using a different batch of strips after storage at RT for 0 and 6 months. As shown in Table 2, 8 of the 10 tested samples were detected as positive, and 2 of the 10 tested samples were detected as negative. Identical results were observed with strips from the same batch and different batches after different storage times.

**Table 2:** Assessment of the reproducibility and stability of the ICS.

Batch no.	202101		202102		202103	
Month	0	6	0	6	0	6
Positive no.	8	8	8	8	8	8
Negative no.	2	2	2	2	2	2

Three batches of strips (batch numbers: 202101, 202102, and 202103) were used for the detection of PRRSV in 10 virus samples. All strips from each batch were used to test samples two times at a 6-month interval.

### Clinical Application of the ICS

PCR and the ICS were compared with respect to their ability to detect PRRSV in 108 porcine clinical samples. The PCR results identified 46 positive and 62 negative samples, whereas the ICS assay results identified 40 positive samples and 68 negative samples (Table 3). The positive rates of the two methods were 42.59 and 37.03%, respectively. The ICS assay was shown to exhibit relatively high specificity and sensitivity (90.32 and 73.91%, respectively), and relatively high positive predictive value (PPV) and negative predictive value (NPV; 85 and 82.35%, respectively). The overall coincidence rate between the test strip and PCR method was 83.33%, which indicated that the ICS method established in this study can be used for the detection of clinical samples.

**Table 3:** Results of the detection of PRRSV in clinical samples *via* ICS and PCR.

		ICS test		Total	Coincidence
		Positive	Negative		
PCR test	Positive	34	12	46	
	Negative	6	56	62	
	Total	40	68	108	83.33%

## Discussion

PRRSV infection usually occurs in pigs of all ages. The disease is characterized by reproductive failure in sows and respiratory disease in pigs (Albina, 1997). Virus isolation and RT-PCR are reliable methods for the diagnosis of PRRSV infection, but these methods are mostly completed in laboratories with specialized instruments by skilled technicians. The immunochromatographic strip method based on different labeled tracers and PRRSV proteins has been applied to detect PRRSV antigens or antibodies, and among these methods, colloidal gold technology is the most mature (Zhou et al., 2009; Li et al., 2017; Yu et al., 2018). However, due to the limitations of the characteristics of colloidal gold nanoparticles, their stability and sensitivity are lower than those of some new tracers, such as latex microspheres (Li et al., 2019a). Latex microspheres bind with antibodies through covalent coupling, which is more stable than the electrostatic adsorption of colloidal gold nanoparticles. The nanoparticle size range of latex microspheres is markedly larger than that of colloidal gold nanoparticles, and the former thus have a large specific surface area and can bind more antibodies. This fact explains why the stability and sensitivity of latex microspheres are generally better than those of colloidal gold nanoparticles (Li et al., 2019a). The ICS method for detecting PRRSV antigen based on latex microspheres as a tracer has not been reported. In this study, we prepared a monoclonal antibody against the PRRSV N protein that recognized the B-cell epitopes (^107^PTHHTVRLIRVTASPSA^123^) at the C-terminus of the N protein. Previous studies have shown that the 123 ~ 128 amino acid N protein interacts with viral RNA to form the viral nucleocapsid, which is expressed from the smallest subgenomic mRNA (mRNA7) and is the most abundant viral protein expressed in infected cells (Meulenberg et al., 1995a; Dea et al., 2000). N is the most immunogenic viral protein, but anti-N antibodies are non-neutralizing and non-protective (Murtaugh et al., 2002). Previous studies have demonstrated that N is incorporated into virions as disulfide-linked dimers (Mardassi et al., 1996; Wootton and Yoo, 2003), and this dimerization involves Cys 23 (equivalent to Cys 27 in type 1 viruses), which is located within the N-terminal helix (Wootton and Yoo, 2003). Typically, different antibodies that recognize different antigen epitopes are used in ICS for better capture of antigenic proteins. Interestingly, the present study revealed that the C-terminus of the N protein (aa 68 ~ 118) exists in the form of a homodimer using the homology modeling method to predict the 3D structure of the N protein (Figure 3B), which was consistent with a previous study (Doan and Dokland, 2003). Based on structural models, the epitopes recognized by mAb 1H4 were located on both ends of the N protein homodimer (Figure 3C). Based on this background, a latex microsphere-based ICS was constructed to prepare a rapid diagnostic strip for the detection of PRRSV by a single mAb. This novel ICS can be performed for the rapid diagnosis of PRRSV in the field without the need for professional personnel or equipment.

In recent years, an increasing number of studies have reported that the genetic and variant diversity of PRRSV continues to increase in China (Zhang et al., 2018; Jiang et al., 2020; Yu et al., 2020). To date, no relevant study has evaluated the detection ability of an ICS for the current major epidemic NADC30-like strains and potential epidemic NADC34-like strains in China. Therefore, this ICS was assessed for the broad-spectrum detection of different NA-PRRSVs. First, multiple sequence alignments revealed that the N protein epitope recognized by mAb 1H4 contained several substitutions in several strains (Figure 3A). The study also found that mAb 1H4 was fluorescently reactive with representative strains of North American-type PRRSV (data not shown). The applicability results showed that this ICS could still detect North American-type representative strains in China, such as classical strains (CH-1R and MLV), highly pathogenic strains (HuN4, HuN4-F112, TJM-F92, and JXA1-R), NADC30-like strain (SDHS53), and NADC34-like strain (TZJ1341; Figure 6). Therefore, the mutation of some amino acid sites had no effect on the recognition of this epitope by mAb 1H4, which indicated that this epitope is highly conserved. To improve the specificity of the strip test, a mAb was used instead of a polyclonal antibody (pAb) on the test line. The specificity assay revealed that the established test strip exhibits high specificity and no cross-reactivity with other clinical pathogens or EU-PRRSVs. The limit of detection for PRRSV was 5 × 10^2.0^ TCID_50_/ml for the developed test strip, and the limit of detection for SDHS53-N protein was 15 ng/ml, which indicates that this test strip was more sensitive than an ICS developed using a combination of pAb and mAb (limit of detection = 7.8 × 10^3^ TCID_50_/ml; Zhou et al., 2009). However, through the detection of clinical samples, we found that the coincidence rate between this ICS and RT-PCR was relatively lower than that obtained with the method established by Zhou et al. (2009). The specificity and sensitivity of ICS detection were 90.32 and 73.91%, respectively. We speculated that this finding may be due to differences between different sublineage strains. The samples tested by Zhou et al. (2009) were mainly obtained from the epidemic period of a highly pathogenic strain. In recent years, the NADC30-like strain (sublineage 1.8) has replaced the highly pathogenic strain (sublineage 8.7) as the main epidemic strain in China, and its pathogenicity is markedly lower than that of the highly pathogenic strain. The viral load in tissue and serum after infection is relatively lower than that of the highly pathogenic strains (Sun et al., 2016; Zhang et al., 2019; Yu et al., 2021). The NADC30-like strains were the most highly detected strains in the clinical samples tested in this study, which may explain the relatively low coincidence rate. Although PCR is a method with higher sensitivity and a lower detection limit (Table 3), the test strip is both simple and rapid. More importantly, the test strip also exhibited good specificity. Unlike conventional detection methods, latex microsphere-based ICSs can be applied easily without special equipment, requiring only simple visual judgment. Therefore, to make the test results more accurate and digitizable, we will likely use fluorescent microspheres instead of ordinary latex microspheres to further improve the sensitivity and judge negative or positive results through portable detectors and quantitative values.

In summary, the novel ICS based on latex microspheres established in this study can detect PRRSV antigens with high sensitivity and specificity. Moreover, the mAb employed on the strip and the latex microsphere-mAb tracer were ideal for identifying NA-PRRSVs. The ICS has the potential to be employed as a screening tool for the diagnosis of PRRSV infections in the pig industry.

## Conclusion

This study provides the first demonstration of the detection of NA-PRRSV by an ICS based on latex microspheres, which is highly suitable for the detection of PRRSV in the field.

## Data Availability Statement

The original contributions presented in the study are included in the article/supplementary material, further inquiries can be directed to the corresponding author.

## Ethics Statement

The animal study was reviewed and approved by the Committee on the Ethics of Animal Experiments of the Harbin Veterinary Research Institute (HVRI) of the Chinese Academy of Agricultural Sciences (CAAS).

## Author Contributions

QW and WL: conceived and designed the experiments, performed the experiments, and contributed to the writing of the manuscript. ML, HZ, CL, HX, BG, JF, and ZG: contributed reagents or materials and assisted in some experiments. JP, GZ, and ZT: analyzed the data. All authors contributed to the article and approved the submitted version.

## Funding

This work was supported by the Natural Science Foundation of Heilongjiang Province (YQ2019C032) and the Youth Foundation of the State Key Laboratory of Veterinary Biotechnology (SKLVBP202122).

## Conflict of Interest

ML was employed by Beijing IDEXX Yuanheng Laboratories, Co., Ltd.

The remaining authors declare that the research was conducted in the absence of any commercial or financial relationships that could be construed as a potential conflict of interest.

## Publisher’s Note

All claims expressed in this article are solely those of the authors and do not necessarily represent those of their affiliated organizations, or those of the publisher, the editors and the reviewers. Any product that may be evaluated in this article, or claim that may be made by its manufacturer, is not guaranteed or endorsed by the publisher.

## Footnotes

^1^ http://www.swissmodel.expasy.org/
